# Electrospun PLGA–Propolis Scaffolds Regulate Collagen Architecture in Burn Wounds

**DOI:** 10.3390/ijms27115021

**Published:** 2026-06-02

**Authors:** Kinga Orlińska, Paweł Olczyk, Przemysław Motyl, Mateusz Stojko, Krystyna Skalicka-Woźniak, Krzysztof Kamil Wojtanowski, Krzysztof Jasik, Janusz Kasperczyk, Jakub Włodarczyk, Krystyna Olczyk, Jerzy Stojko, Diana Ivanova, Yoana Kiselova-Kaneva, Katarzyna Komosińska-Vassev

**Affiliations:** 1 Department of Community Pharmacy, Faculty of Pharmaceutical Sciences in Sosnowiec, Medical University of Silesia in Katowice, 8b Jedności, 41-205 Sosnowiec, Poland; 2 Faculty of Medical Sciences and Health Sciences, Radom University, Chrobrego 27, 26-600 Radom, Poland; p.olczyk@urad.edu.pl; 3 Faculty of Mechanical Engineering, Radom University, Stasieckiego 54, 26-600 Radom, Poland; 4 Centre of Polymer and Carbon Materials, Polish Academy of Sciences, M. Curie-Skłodowskiej 34, 41-819 Zabrze, Poland; mstojko@cmpw-pan.edu.pl (M.S.); jkasperczyk@cmpw-pan.pl (J.K.); jwlodarczyk@cmpw-pan.pl (J.W.); 5 Department of Chemistry of Natural Products, Medical University of Lublin, Chodźki 1, 20-093 Lublin, Poland; kskalicka@pharmacognosy.org; 6 Department of Pharmacognosy, Medical University of Lublin, Chodźki 1, 20-093 Lublin, Poland; krzysztof.wojtanowski@umlub.pl; 7 Wladyslaw Bieganski Collegium Medicum, Jan Dlugosz University in Czestochowa (UJD), Armii Krajowej 13/15, 42-200 Częstochowa, Poland; k.jasik@ujd.edu.pl; 8 Department of Biopharmacy, Faculty of Pharmaceutical Sciences in Sosnowiec, Medical University of Silesia in Katowice, Jedności 8, 41-200 Sosnowiec, Poland; 9 Department of Clinical Chemistry and Laboratory Diagnostics, Faculty of Pharmaceutical Sciences in Sosnowiec, Medical University of Silesia in Katowice, 8 Jedności, 41-200 Sosnowiec, Poland; olczyk@sum.edu.pl (K.O.); kvassev@sum.edu.pl (K.K.-V.); 10 Department of Toxicology and Bioanalysis, Faculty of Pharmaceutical Sciences in Sosnowiec, Medical University of Silesia in Katowice, Ostrogórska 30, 41-200 Sosnowiec, Poland; jstojko@sum.edu.pl; 11 Department of Biochemistry, Molecular Medicine and Nutrigenomics, Faculty of Pharmacy, Medical University “Prof. Dr. Paraskev Stoyanow” of Varna, 55 Marin Drinov Street, 9002 Varna, Bulgaria; divanova@mu-varna.bg (D.I.); yoana.kiselova@mu-varna.bg (Y.K.-K.)

**Keywords:** burn wounds, collagen architecture, electrospinning, PLGA scaffold, propolis

## Abstract

Wound (especially burn) healing, is a complex process involving cells e.g., leukocytes, macrophages, keratinocytes, fibroblasts, endothelial cells and platelets. Additionally, glycosaminoglycans, proteoglycans and collagen participate in the remodeling of the extracellular matrix (ECM). It is well-known that collagen, especially type I and III, are products of fibroblasts. These cells proliferate in the final phase wound healing. The various stages of skin regeneration, i.e., processes such as hemostasis, inflammation, cells’ growth, differentiation and migration, can be accelerated by certain natural, biologically active, factors. The aim of this study was to evaluate the effect of a propolis-incorporated nonwoven on collagen organization and tissue architecture in a porcine burn model. Propolis, a bee-product rich in numerous diverse phenolic compounds, has been used since ancient times for the treatment of skin diseases and repairing various types of wounds. The research material consisted of tissue sections taken from burn wound beds inflicted in domestic pigs. The samples of skin sections were evaluated using routine light microscopy. Also, ultrastructural studies have been performed with scanning electron microscopy and atomic force microscopy. Nonwovens were made by electrospinning of a poly(lactide-co-glycolide) 85:15 copolymer (PLGA) without active compound as well as containing 5 wt% and 10 wt% of propolis. Electrospining solutions were dissolved in 1,1,1,3,3,3-hexafluoro-2-propanol (HFIP). In the burn wound bed, propolis-incorporated dressings were associated with improved epithelial and dermal tissue organization and with an increased presence of organized collagen bundles (without direct assessment of collagen subtypes). These observations suggest that propolis incorporation is associated with improved collagen organization and tissue architecture at the histological level. The enhanced collagen deposition and organization observed in wounds treated with propolis-containing nonwovens indicate improved extracellular matrix remodeling and structural tissue organization in burned skin.

## 1. Introduction

Wound healing (WH), including burn healing, is a dynamic, multi-stage physiological process that restores tissue homeostasis through hemostasis, inflammation, proliferation, and remodeling. These phases are regulated by coordinated activity of various cells (e.g., leukocytes, macrophages, keratinocytes, fibroblasts, endothelial cells, and platelets) and extracellular matrix (ECM) components, including glycosaminoglycans (GAGs), proteoglycans (PGs), and collagens (Colls) [1,2,3].

Burn injuries remain a major global health problem, with millions of patients requiring medical care annually [4,5]. Their treatment is particularly challenging due to prolonged inflammation, increased susceptibility to infection, and excessive scarring [6,7,8]. Chronic wounds disrupt ECM remodeling, where persistent inflammation, hypoxia, and metalloproteinase activity impair the balance between matrix synthesis and degradation, leading to disorganized collagen architecture and impaired angiogenesis [6,7,8,9].

Current therapeutic strategies increasingly focus on biomaterial-based systems that actively support tissue regeneration rather than only providing protection [6,7,8,9]. Electrospun fibrous scaffolds are especially promising due to their high porosity, ability to support cell adhesion and migration, and capacity to deliver bioactive compounds while ensuring gas exchange [6,10,11,12]. Additionally, their elasticity and flexibility allow adaptation to dynamic wound environments [13,14,15,16].

ECM components such as GAGs, PGs, and collagens play essential roles in cell signaling, migration, and scar formation [17]. Advanced electrospun dressings incorporating bioactive substances, including propolis, may reduce abnormal scarring while promoting regeneration [13,14,15,18,19].

Propolis, a resinous substance collected by Apis mellifera, contains flavonoids, phenolic acids, and other bioactive compounds with antibacterial, anti-inflammatory, antioxidant, and regenerative properties [19,20,21,22,23]. These compounds influence collagen deposition and organization, which is critical for ECM integrity [16,23,24,25,26,27,28,29,30,31]. Moreover, collagen modulates fibroblast and keratinocyte activity, thereby supporting tissue regeneration [29,31,32].

Despite increasing interest in propolis-based biomaterials, in vivo evidence regarding their influence on collagen organization and extracellular matrix remodeling in burn wounds remains limited, particularly in clinically relevant large-animal models. Therefore, the aim of the present study was to evaluate the effects of electrospun nanofibrous dressings containing propolis (5% and 10%) in a porcine burn model, with particular emphasis on collagen deposition, organization, and tissue architecture. This study provides novel insights into the role of propolis-incorporated scaffolds in modulating extracellular matrix organization in burned tissue.

It should be noted that the present study focuses on morphological and ultrastructural evaluation of collagen organization, without direct molecular assessment of collagen type I/III expression.

## 2. Results

Histological comparison of skin sections taken from wounds inflicted in four experimental animals [Wounds no. I. (physiological saline-treated wounds, isolated immediately after the burn); Wounds no. II. (treated with a sterile dressing); Wounds no. III. (treated with 5% propolis nonwoven dressing); Wounds no. IV. treated with 10% propolis nonwoven dressing)] allowed to notice clear disparities. All tissue samples isolated from burn wounds proved the skin’s intense modification.

Progressive differences in tissue organization and collagen architecture were observed between experimental groups depending on the applied treatment. The observations are based on qualitative histological and ultrastructural assessment without quantitative measurements. To support qualitative observations, a semi-quantitative analysis of selected histological features was performed. A higher number of blood vessels and hair follicles per field was observed in propolis-treated groups compared with controls. Representative microscopic fields were selected based on typical morphological features observed consistently across samples within each experimental group. All samples were collected, processed, stained, and evaluated according to the same experimental and histological protocol across all groups, enabling direct qualitative comparison of morphological features.

### 2.1. Wounds No. I. (Tissue Samples Taken Directly After Burn)

**Figure 1:**
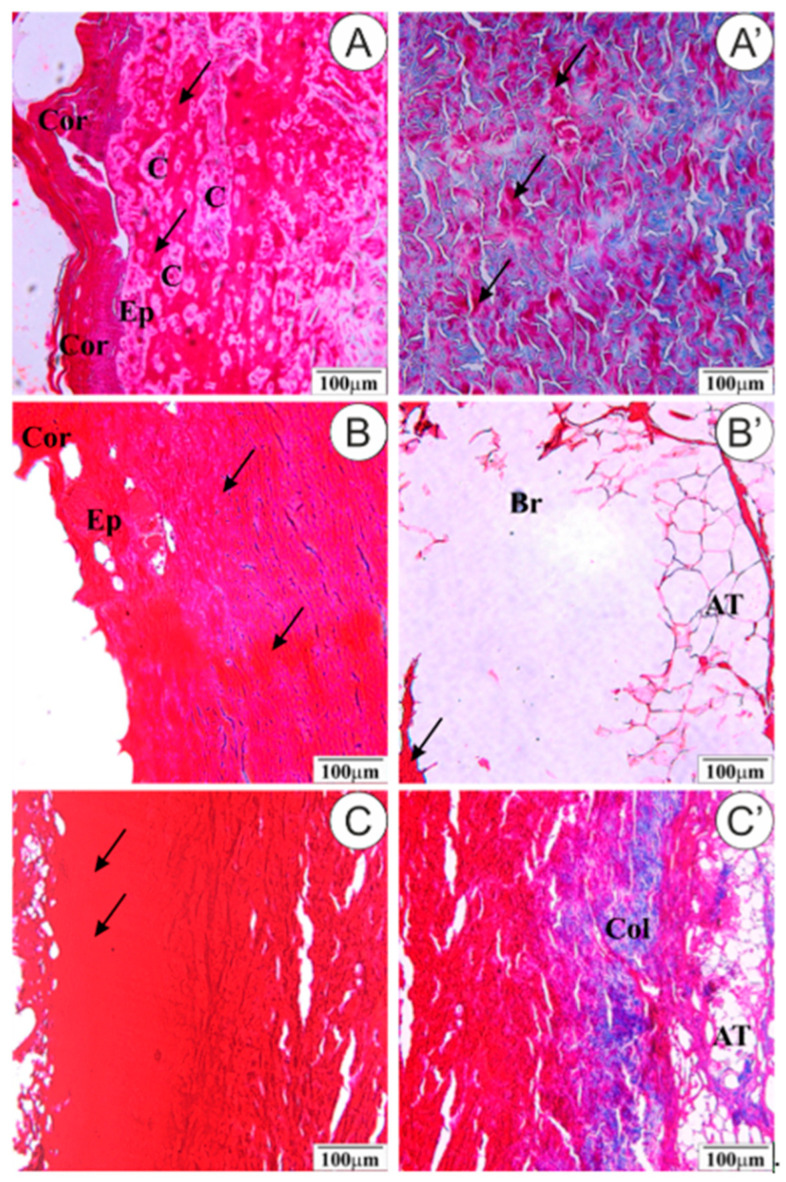
Dynamics of skin changes immediately after burns and regenerative processes of untreated tissues on the 3rd and 21st day. (**A**) Skin cross-section, a superficial layer (wounds no. I.—immediately after burning with high temperature). Arrows—amorphous material lacking the cell outlines, C—the cells damaged as induced by high temperature, Cor—stratum corneum, Ep—epidermis. (**A′**) Skin cross-section, a deep zone (Wounds no. I.—immediately after burning with high temperature). Arrows—amorphous masses of damaged cells, cytoplasm stained with azocarmine, between collagen fibers, stained blue. (**B**) Skin cross-section, a superficial layer; third day after scald (wounds no. I., 3rd day after burn). Arrows—destroyed cell fragments between collagen fibers, intensely stained with azocarmine, Cor—stratum corneum, Ep—epidermis. (**B′**) Transverse section of burned skin (deeper situated zone), the third day after burn (wounds no. I., 3rd day after burn). Arrow—the formless masses of destroyed cells, intensely stained with azocarmine, AT—adipose tissue, Br—damaged dermis tissue. (**C**) Transverse section of burned pig’s skin—superficial layer (wounds no. I., 21st day of the experiment). Arrows—destroyed cells. Histological section stain with the AZAN method. Scale bar: 100 µm; (**C′**) Transverse section of burned pig’s skin—deeper layer (wounds no. I., 21st day of the experiment). AT—adipose tissue, Col—the collagen stained with aniline blue. Histological section stain with the AZAN method. Scale bar: 100 µm.

The initial effect of high-temperature impact on skin wounds no. I. was typically superficial, limited to the epidermis and outer layers of the dermis areas. The stratum corneum was completely or partially broken, with blistering, while epidermal cells formed a necrotic mass. Only a few spots exhibited groups of cells with a pyknotic nucleus and non-staining cytoplasm. Most of the layer was a formless mass stained with azocarmine [Figure 1A].

The affected tissue in the initial period did not show any changes within deeper layers of the skin. The area between the exterior layers and the subcutaneous layer with adipose tissue contained a fair amount of fully arranged fibers that were stained in blue using an AZAN method [Figure 1A′].

Figure 1 presents the dynamics of regenerative changes after burns in group I, immediately after the burn (1A, 1A′), on day 3 (1B, 1B′), and on day 21 (1C, 1C′). Microphotographs show the regenerative potential of the epidermis and dermis of the skin after burns without the use of factors supporting the regeneration processes. Group I is therefore the control group.

**Figure 2:**
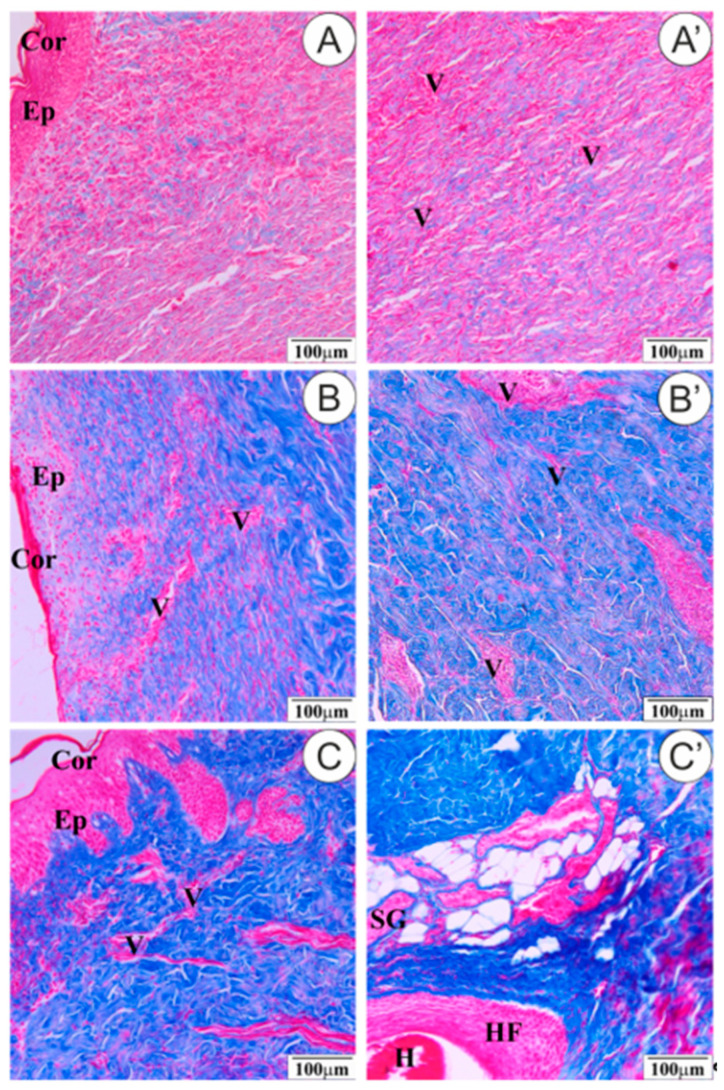
Regenerative changes in the skin 21 days after a burn using various treatment methods. (**A**) Skin cross-section, a superficial layer (wounds no. II, treated with a sterile dressing, 21st day of the experiment). Ep—epidermis, Cor—corneum layer. Histological section stain with the AZAN method. Scale bar: 100 µm; (**A′**) Pig’s skin cross-section, a deeper layer (wounds no. II, treated with a sterile dressing, 21st day of the experiment). (**B**) Skin cross-section, a superficial layer (wounds no. III. (21st day after burn, treated with 5% propolis nonwoven). Ep—epidermis, Cor—corneum layer, V—blood vessel. Histological section stain with the AZAN method. Scale bar: 100 µm; (**B′**) Pig’s skin cross-section, a deeper layer (wounds no. III, 21st day after burn, treated with 5% propolis nonwoven). (**C**) Skin cross-section, a superficial layer (wounds no. IV., 21st day after burn, treated with 10% propolis nonwoven). Ep—epidermis, Cor—corneum layer, V—blood vessel. Histological section stain with the AZAN method. Scale bar: 100 µm; (**C′**) Pig’s skin cross-section, a deeper layer (wounds no. IV., 21st day after burn, treated with 10% propolis nonwoven). H—cross-section through hair, HF—hair follicle, SG—sebaceous gland. Histological section stained with the AZAN method. Scale bar: 100 µm.

The key importance in terms of photographic documentation regarding the effectiveness of bioactive factors (Table 1) in supporting the regeneration of burn wounds, in accordance with the research hypothesis, is presented in Figure 2’s microphotographs.

### 2.2. Wounds No. I. (Tissue Samples Taken on the 3rd Day After Burn)

Tissue samples isolated on the 3rd day after burn had a deeper burn effect than those taken directly after burn. Histological slides showed only a formless mass with no remains of cells in the epidermis [Figure 1B].

Moreover, there were necrotic features accompanied by the debris of cells, along with a formless and fibrous mass containing empty spaces. Fibril elements of both the dermis and subcutaneous layer were stained with an intense blue color. Narrow bands of collagen fibers were visible around the destroyed cytoplasmic masses and adipocytes [Figure 1B′].

### 2.3. Wounds No. I. (Tissue Samples Taken on the 21st Day After Burn)

The analyses involving a histological assessment of skin’s cross-section samples isolated on the 21st day after burn revealed significant changes. Histological analyses showed structural features consistent with tissue remodeling three weeks after burn induction. The necrotic changes of the outer layer were still abundant; however, cells were also noted as a sign of epidermis’ renewal [Figure 1C]. Cell proliferation, increased presence and organization of collagen fibers, and restoration of the skin’s zonation were further evidence of histological tissue reorganization. In general, the most evident structural improvement was noted in the dermis and subcutaneous tissue [Figure 1C′].

### 2.4. Wounds No. II. (Taken on the 21st Day After Burn, Treated with a Sterile Dressing PLGA Without Propolis)

The observations of skin’s renewal were even more obvious in the second group of animals. First of all, keratinized stratified squamous epithelium was correctly formed in the superficial layer [Figure 2A]. Moreover, there were a lot of pink-stained cells and correctly stained blue fibers. A large number of small blood bud vessels were visible [Figure 2A′].

Compared to untreated wounds, the use of a sterile dressing resulted in improved tissue organization, partial re-epithelialization, and increased vascularization. However, the histological pattern of tissue organization remained incomplete and less ordered compared with the propolis-treated groups.

### 2.5. Wounds No. III. (21st Day After Burn, Treated with 5% Propolis Nonwoven)

The analyzed material collected from animals showed a large number of cells in both superficial [Figure 2B] and deeper [Figure 2B′] layers. Features such as cellular proliferation, restoration of the skin zonation, and tight arrangement of blue-stained collagen fibrils are consistent with improved tissue organization.

The application of the 5% propolis dressing was associated with improved tissue organization, as evidenced by increased cellular proliferation, improved collagen fiber organization (based on morphological assessment), and a more distinct zonal structure of the skin compared with the control groups.

### 2.6. Wounds No. IV. (21st Day After Burn, Treated with 10% Propolis Nonwoven)

The skin samples revealed a well-formed keratinized multilayer squamous epithelium [Figure 2C]. In the analyzed skin samples, blue-stained collagen fibrils accompanied by numerous blood capillaries were observed. Additionally, hair follicles and sebaceous glands were visible in skin samples’ cross sections [Figure 2C′].

The 10% propolis dressing showed the most advanced structural features at the histological level, including well-organized collagen fibers, restored epidermal structure, increased vascularization, and the presence of skin appendages.

A qualitative trend toward improved tissue organization was observed across all experimental groups. Consistent morphological differences were identified between groups, indicating progressively more organized histological features in propolis-treated wounds. The degree of histological tissue organization increased from untreated wounds, through sterile dressings, to the propolis-treated groups, with the most organized appearance observed in the 10% propolis scaffold group. The present study does not provide direct evidence of accelerated wound closure or wound size reduction, as macroscopic wound documentation and quantitative wound closure analysis were not performed.

To perform a semi-quantitative analysis of representative areas of observation on histological sections, histological fields stained with the AZAN method, an additional qualitative assessment of collagen deposition and organization was performed. Collagen-positive areas were identified based on their blue-blue-violet color, indicating the affinity of the test material for aniline blue. The degree of staining was determined using fixed color thresholds, uniform for all analyzed slides.

**Figure 3:**
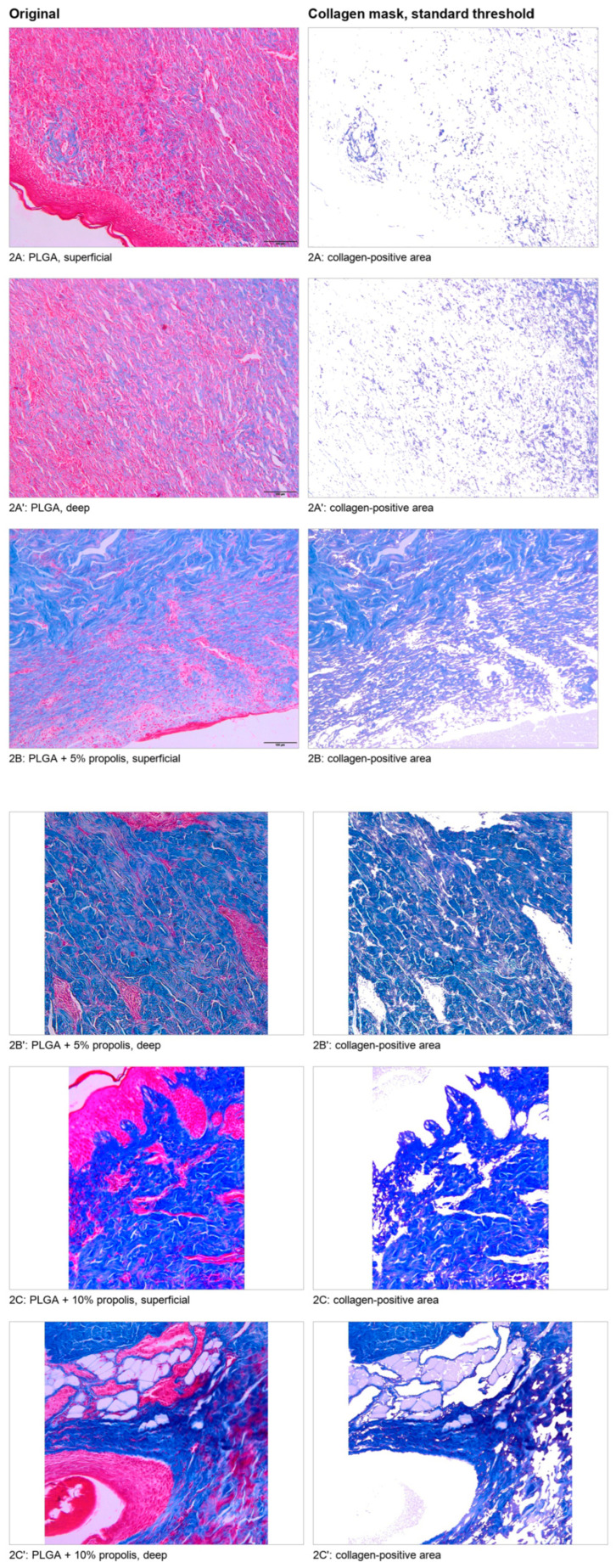
Semi-quantitative analysis of the observed areas of the slides presented in Figure 2. (In Figure 3, the numbering of individual slides was maintained to emphasize which slides were used for the semi-quantitative analysis).

For quality control, masks were generated in which pixels classified as collagen-positive were left visible, while the remainder of the image was set to white [Figure 3].

Semi-quantitative analysis of representative histological fields stained with the AZAN method showed higher percentages of collagen-positive areas in the groups treated with PLGA dressings containing propolis than in the group treated with PLGA dressings without propolis. In Figure 3, using the standard thresholding variant, the mean collagen fraction was 9.59% in the PLGA without propolis group, 76.65% in the PLGA + 5% propolis group, and 62.94% in the PLGA + 10% propolis group (Table 2).

A similar effect was observed after using a more sensitive and restrictive thresholding variant, which supports the credibility of the observed results. They indicate that the intensity of collagen synthesis was significantly higher in the analyzed preparations from the groups treated with propolis-containing dressings.

### 2.7. Results Verification—Scanning Electron Microscope (SEM) and Atomic Force Microscope (AFM)

**Figure 4:**
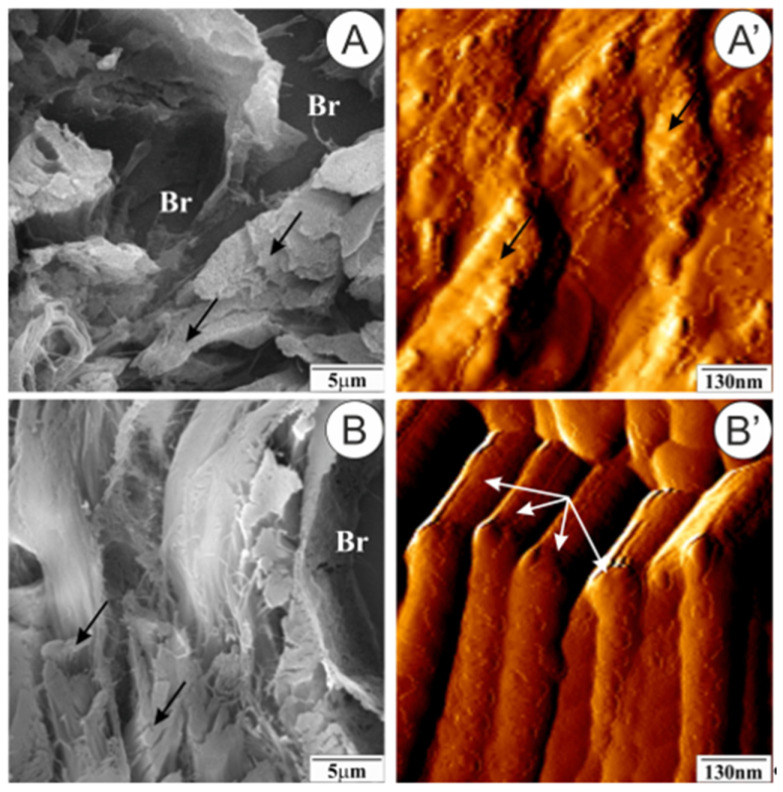
Changes after burns on the 3rd day (without treatment) and on the 21st day after applying a dressing with 10% propolis, summarizing the image of the dermis structure. (**A**) Transverse section of burned skin: deeper zone of the dermis and subcutaneous layer, the third day after burn (wounds no. I.). Arrows—bundles of collagen fibrils changed conformation with high temperature, Br—break of tissue. SEM. Scale bar: 5 µm; (**A’**) Skin section, a deeper layer of the dermis: the third day after burn (wounds no. I.). Arrows—damaged collagen fibrils. AFM. Scale bar: 130µ. (**B**) Section of pig’s skin (wounds no. IV., 21st day after burn, treated with 10% propolis nonwoven). Arrows—bundles of collagen fibrils. SEM. Scale bar: 5 µm; (**B’**) Section of pig skin (wounds no. IV., 21st day after burn, treated with 10% propolis nonwoven). Arrows—bundles of collagen fibrils are forming plates. AFM. Scale bar: 130 µm.

The spreads of skin tissues and changes in fibril structure were documented by scanning electron microscope and atomic force microscope. These studies were performed to confirm the routine histological observations and the description derived only from trichrome-stained reactions [Figure 4A,A′]. The described observations confirmed the destruction of collagen fibers and cell deformations.

The collagen fibers were closely spaced, forming thicker bundles. Research conducted with a scanning electron microscope allowed observation of fiber bundles in both dermis and subcutaneous tissues. Representative areas were selected based on consistent structural features observed across samples. [Figure 4B]. Analyses using AFM additionally showed characteristic arrangements of collagen fibrils. Bundles of fibers arranged in parallel have formed distinctive plates [Figure 4B′].

The observed regularity and order of collagen fibers may be associated with the mechanical properties and elasticity of the electrospun dressing. The presence of a flexible yet structurally stabilizing support layer may contribute to more uniform mechanical conditions in the wound bed, thus contributing to the formation of a new, ordered arrangement of collagen fibers [16,29,31].

These ultrastructural observations further suggest that propolis-based dressings may contribute to improved collagen organization and structural integrity of the analyzed tissue compared with untreated conditions.

### 2.8. Chemical Composition of Propolis (LC-MS Analysis)

**Figure 5:**
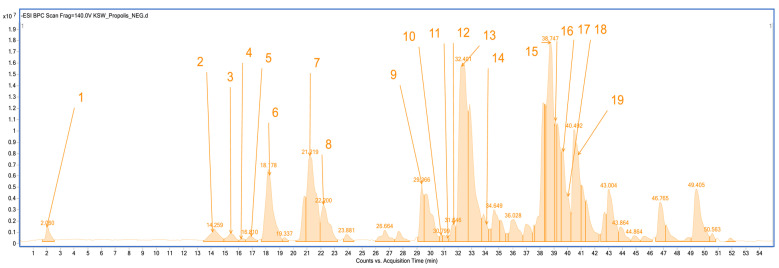
The BPC chromatogram is an illustration for Table 3 where all identified compounds are marked.

Results of LC-MS of the methanolic extract of propolis are shown in Table 1. Nineteen compounds were tentatively identified in negative mode (Figure 5). Flavonoids, free phenolic acids, and phenolic acid esters were the main groups of compounds. As dominant pinobanksin, pinostrobin, chrysin, and pinocembrin were indicated.

**Table 3:** ESI-QTOF-MS analysis of the methanolic extract of Propolis.

No	Tentative Assignment	Retention Time [min]	Formula	Molecular Ion [m/z]	MS/MS Fragments [m/z]
1	Saccharose	2.060	C_6_H_12_O_6_	179.0566	89.0242; 71.0140; 59.0142
2	Hydroxybenzoic acid isomer	14.337	C_7_H_6_O_3_	137.0247	108.0209; 92.0258; 81.0347; 65.0032; 53.0394
3	Hydroxybenzoic acid isomer	15.354	C_7_H_6_O_3_	137.0247	93.0346; 65.0401
4	Vanillin	16.105	C_7_H_6_O_2_	151.0403	108.0214
5	Benzoic acid	16.438	C_8_H_8_O_3_	121.0277	92.0270
6	Caffeic acid	17.940	C_9_H_8_O_4_	179.0349	135.0442; 107.0497; 89.0391
7	Coumaric acid	20.941	C_9_H_8_O_3_	163.0393	119.0503; 93.0346
8	Ferulic acid	21.991	C_10_H_10_O_4_	193.512	178.0267; 149.0608; 134.0369
9	Ethyl Caffeate	29.362	C_11_H_12_O_4_	207.0668	179.0342; 161.0237; 134.0369
10	Quercetin	30.762	C_15_H_10_O_7_	301.0369	178.9993; 151.0031; 121.0293; 107.010; 93.0351; 83.0143
11	Luteolin	30.896	C_15_H_10_O_6_	399.1107	199.0409; 175.0406; 133.0287; 107.0134
12	Isorhamnetin	31.680	C_16_H_12_O_7_	315.0508	300.0265; 271.0238; 255.0292; 243.0296
13	Pinobanksin	32.113	C_15_H_12_O_5_	271.0617	253.0498; 225.0552; 197.0599; 161.0601; 151.0031; 125.0234; 115.0548; 107.0133
14	Apigenin	33.898	C_15_H_10_O_5_	269.0462	197.0599; 151.0026; 117.0340; 107.0128
15	Dimethylallyl caffeate	38.867	C_14_H_16_O_4_	247.0979	179.0348; 134.0377
16	Pinostrobin	39.201	C_16_H_14_O_4_	269.0820	161.0236; 134.0366; 106.0420
17	Chrysin	39.534	C_15_H_10_O_4_	253.0513	213.0548; 171.0450; 143.0497; 107.0136
18	Pinocembrin	39.868	C_15_H_12_O_4_	255.0657	213.0556; 171.0447; 151.0031; 145.0659; 107.0137
19	Caffeic acid phenethyl ester (CAPE)	41.118	C_17_H_16_O_4_	283.0974	179.0341; 161.0236; 135.0448

Semiquantification of main hydroxycinnamic acid derivatives and flavonoids was performed, and the results are presented in Table 4.

### 2.9. Morphology of Electrospun Nonwovens (SEM)

Scanning electron microscopy revealed that all three electrospun formulations: PLGA 85/15, PLGA 85/15 with 5 wt% propolis, and PLGA 85/15 with 10 wt% propolis, produced continuous, non-woven fibrous mats with randomly oriented fibers and no macroscopic defects (Figure 5). The pure PLGA 85/15 mat exhibited a well-defined fibrous structure on both the inner and outer surfaces, with smooth, cylindrical fibers. The mean fiber diameter was 0.9985 ± 0.1229 µm (*n* = 50), reflecting a relatively narrow and uniform diameter distribution.

Incorporation of propolis at 5 wt% preserved the continuous fibrous architecture but was accompanied by a slight reduction in mean fiber diameter to 0.8183 ± 0.101 µm (*n* = 50). Individual fibers remained smooth and cylindrical with no surface porosity or particulate inclusions, and fiber junction points showed simple overlapping contacts.

**Figure 6:**
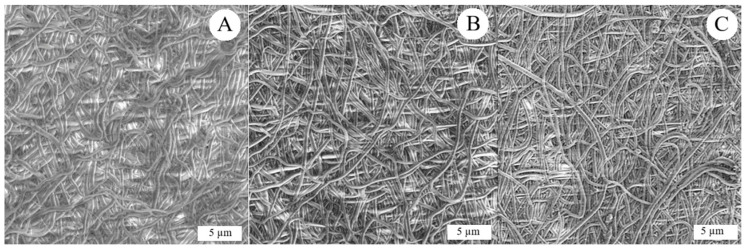
SEM images of the nonwoven materials obtained by electrospinning (5000×); (**A**) PLGA 85/15; (**B**) PLGA 85/15 containing 5 wt% of propolis, and (**C**) PLGA 85/15 containing 10 wt% of propolis. Scale bar: 5 µm.

Further reduction in mean fiber diameter was observed with the addition of 10 wt% propolis, yielding a mean diameter of 0.7419 ± 0.105 µm (*n* = 50). The overall trend of decreasing fiber diameter with increasing propolis content suggests a propolis-dependent modification of solution viscosity and/or surface tension during electrospinning, facilitating greater jet elongation and thinning. Despite the reduction in diameter, all three formulations maintained a three-dimensional porous fibrous architecture expected to support cell attachment and infiltration (Figure 6).

## 3. Discussion

The primary aim of the present study was to evaluate and compare the effects of nanofibrous dressings containing propolis as an active substance, in two different concentrations (5% and 10%), on collagen organization and tissue architecture. This was achieved by qualitatively assessing collagen organization and distribution within the matrix of the burn wounds using an in vivo model involving Polish domestic pigs.

The choice of this animal model is justified by the remarkable similarities between porcine and human skin in anatomical, physiological, and biochemical terms. These similarities include comparable thickness and structure of the epidermis and dermis, the structure of the connection between the epidermis and the skin proper, the structure of subcutaneous tissue, and the density and distribution of blood vessels. Furthermore, pigs and humans share similar epithelial cell proliferation rates, keratin protein profiles, stratum corneum lipid composition, and collagen fiber orientation [33,34].

To isolate the therapeutic effects of propolis, the study utilized two control groups: a saline-treated group reflecting baseline healing and a propolis-free nanofiber group to account for the mechanical impact of the nonwoven matrix. Our findings show that collagen organization in the burn wound bed appears to be dependent on the type of dressing used, highlighting the role of bioactive scaffolds in modulating extracellular matrix organization during tissue repair.

The latter process, requiring precise interaction of multiple cell types and various types of molecules, encompasses hemostasis, inflammation, biosynthesis, and remodeling [35]. Inflammation, induced already during hemostasis, develops on the first day after the injury and remains particularly severe until the third day if the repair process is progressing properly. The primary participants in this stage are initially neutrophils, and then—in its advanced phase—lymphocytes and monocytes/macrophages [36].

Beyond its influence on phagocytosis and macrophage migration, propolis acts as a potent stimulator of the regenerative phase of tissue repair. This pleiotropic natural substance may also influence collagen deposition and accumulation in the newly formed matrix of damaged tissue [37]. In addition to stimulating collagen production, propolis has been shown to regulate fibroblast activity by enhancing their proliferation and metabolic activity, which accelerates the formation of granulation tissue. Furthermore, certain propolis constituents, particularly caffeic acid phenethyl ester (CAPE), may promote the differentiation of fibroblasts into myofibroblasts, thereby influencing tissue remodeling while simultaneously limiting excessive fibrosis and hypertrophic scar formation [1,38,39,40].

In addition to the structural effects observed in the present study, propolis may influence wound healing through multiple biological mechanisms that were not directly evaluated here. Previous studies have demonstrated that propolis exhibits significant anti-inflammatory and regenerative properties, which are associated with the modulation of key signaling pathways involved in tissue repair [38,41]. These effects include the regulation of cellular responses to oxidative stress and the attenuation of processes leading to extracellular matrix degradation, which are particularly important in the context of burn-induced tissue injury.

Furthermore, propolis has been shown to influence fibroblast activity, including enhancement of their metabolic activity, proliferation, and involvement in collagen synthesis and remodeling [39,40]. These effects may contribute to extracellular matrix formation and may contribute to improved structural organization of collagen fibers observed in the present study.

In addition, bioactive compounds present in propolis, such as flavonoids (e.g., quercetin), have been reported to regulate key molecular pathways involved in wound healing, including those related to fibroblast activation, angiogenesis, and growth factor expression (e.g., VEGF and TGF-β) [42]. These mechanisms further support the role of propolis in promoting regenerative processes.

Moreover, propolis and its components have been associated with improved re-epithelialization and with broader wound healing responses reported in previous studies, which may involve stimulation of epidermal cell activity and restoration of skin integrity [43,44]. These processes are important for tissue repair and restoration of skin integrity. However, these mechanisms were not directly assessed in the present study.

Although these mechanisms were not directly investigated in the present study, they may partially explain the enhanced collagen organization and improved tissue architecture observed in the propolis-treated groups. Future studies should focus on detailed molecular and cellular analyses to further elucidate the role of propolis in modulating key processes involved in burn wound healing.

The above observations are further supported by the results of the present study. The application of the 5% propolis scaffold over three weeks was associated with the presence of numerous cells in both superficial and deeper layers of the analyzed tissue samples. This was accompanied by active cellular proliferation, restoration of the skin zonation, and dense arrangement of collagen fibrils, which is consistent with improved histological tissue organization. Our findings align with the observations of Rojczyk et al. [43], who indicated that propolis enhances the proliferative phase by stimulating keratinocyte formation and supporting tissue repair [38]. It is worthy of note that a three-week treatment of thermal injuries with 10% propolis scaffold demonstrated, among others, properly organized keratinized multilayer squamous epithelium. Comparable (although not fully analogous) research outcomes of Jastrzębska-Stojko et al. [45] showed a stimulatory effect of pharmaceutical formulations containing propolis and honey, respectively, on the formation of granulation tissue as well as the creation and organization of the epithelium.

The experimental procedures conducted in this study revealed that tissue samples treated with a 10% propolis dressing exhibited collagen fibers in close association with developing blood vessels, hair follicles, and sebaceous glands.

These qualitative changes in collagen organization suggest that the applied bioactive scaffold may influence extracellular matrix remodeling and tissue organization. The quantitative analysis presented here should be considered semi-quantitative and exploratory in nature and interpreted accordingly. Importantly, the present study does not include direct assessment of wound closure dynamics or macroscopic wound size reduction, and the findings should therefore be interpreted within the context of histological and ultrastructural observations. Such a balanced regulation of collagen fiber architecture is consistent with previously reported mechanisms of propolis action and highlights the synergistic role of scaffold structure and bioactive compound release in guiding functional tissue regeneration [6,10]. Additionally, propolis has been reported to modulate extracellular matrix turnover through the regulation of matrix metalloproteinase activity. Experimental studies demonstrated that propolis has been reported to reduce the expression of collagen-degrading enzymes such as MMP-2 and MMP-9, which are frequently elevated in chronic wounds and contribute to the degradation of collagen types I and III. Simultaneously, propolis has been reported to increase the levels of tissue inhibitor of metalloproteinases (TIMP-2), thereby maintaining the balance between collagen synthesis and degradation and protecting newly formed collagen fibers from excessive breakdown [38,40,41,46,47].

Importantly, these findings indicate that propolis may support collagen deposition but also protects newly formed collagen from premature degradation within the wound matrix. These qualitative changes in collagen organization suggest that the applied bioactive scaffold may influence extracellular matrix remodeling. Such a balanced regulation of collagen fiber architecture is consistent with previously reported mechanisms of propolis action and highlights the synergistic role of scaffold structure and bioactive compound release in guiding functional tissue regeneration [6,10]. Limiting excessive metalloproteinase activity and restoring the physiological MMP/TIMP balance, propolis supports the stabilization and accumulation of newly synthesized collagen fibers in the regenerating tissue. This dual mechanism—simultaneous stimulation of collagen production and protection of the newly formed matrix—appears to be a key factor contributing to the improved structural organization of the extracellular matrix observed in propolis-treated tissue [38,40,48].

The histological pattern observed in the present study is consistent with previous reports suggesting that propolis and its bioactive constituents may influence collagen accumulation, fibroblast activity, and extracellular matrix remodeling during wound healing [40,49,50,51,52,53,54]. Previous studies in burn wound models have described the effects of propolis on extracellular matrix components, including collagen type I and III, as well as glycosaminoglycans.

However, it should be emphasized that the present study primarily evaluates the morphological appearance of collagen using AZAN staining and does not directly assess collagen subtype expression or the activity of molecular signaling pathways involved in extracellular matrix remodeling.

Therefore, the most appropriate interpretation is that PLGA dressings containing propolis were associated with a greater visible fraction of collagen-positive areas and a more organized extracellular matrix architecture within the analyzed histological fields. However, these findings do not determine whether the observed effect resulted from increased collagen synthesis, reduced degradation, accelerated fiber maturation, altered spatial organization, or changes in the relative proportion of collagen type I to type III.

From the perspective of tissue regeneration, the mechanical properties of the dressing may additionally influence cellular behavior and extracellular matrix remodeling. Elastic scaffolds have been reported to support fibroblast adhesion and migration, while limiting excessive mechanical stress within the wound bed [16,29,31]. Consequently, mechanically compliant dressings provide a stabilized environment that fosters organized collagen deposition and improved structural maturation of the newly formed tissue [16,30,31].

Poly(lactide-co-glycolide) (PLGA) copolymers were selected as scaffold-forming materials due to their well-documented biocompatibility and predictable degradation behavior [10]. These materials are also widely recognized for their tunable mechanical properties, which can be adjusted by varying copolymer composition and processing parameters [12,14]. Electrospun PLGA nonwovens exhibit a balance between mechanical stability and elasticity, ensuring sufficient resistance to handling and application, while maintaining the flexibility required for prolonged contact with regenerating tissue. Such mechanical properties are essential for maintaining dressing integrity throughout the period of tissue repair [10,14].

The fiber morphology and diameter trends observed in the present study are consistent with the research group findings reported in a manuscript widely characterizing PLGA—propolis nonwovens [10], who fabricated electrospun nonwovens from the same PLGA 85/15 copolymer with 5 wt% and 10 wt% propolis under comparable electrospinning conditions. In that study, SEM analysis confirmed a regular, smooth, and elongated fiber structure without macroscopic deformation for all three formulations, which is in agreement with the morphology documented here. The progressive reduction in fiber diameter observed in the present work with increasing propolis content is mechanistically consistent with the potential plasticizing effect of propolis on the PLGA matrix reported by the authors [10], who demonstrated a decrease in glass transition temperature (Tg) from 57 °C for neat PLGA 85/15 to 54 °C and 53 °C for the 5 wt% and 10 wt% propolis-containing variants, respectively. This reduction in Tg reflects increased chain mobility in the presence of propolis, which likely lowers solution viscosity and facilitates greater jet elongation during electrospinning, resulting in thinner fibers at higher additive concentrations.

By adjusting the ratio of lactidyl to glycolidyl units, it is possible to tailor both the mechanical stability of the electrospun scaffold and the kinetics of bioactive compound release, which is particularly advantageous in the treatment of deep burn wounds requiring prolonged structural support [8].

The phenomena of collagen accumulation, angiogenesis, and restored dermal architecture, characterized by the presence of hair follicles and sebaceous glands, all of which are stimulated by propolis action, have been documented by other authors [55,56,57].

In addition to these effects, propolis exhibits strong anti-inflammatory properties and has been shown to inhibit signaling pathways such as NF-κB, p38 MAPK, and ERK, which are involved in the induction of inflammatory mediators and collagen-degrading enzymes [38,40]. Through this mechanism, propolis not only promotes collagen synthesis but also protects the newly formed extracellular matrix from inflammatory degradation processes [38,40,46]. Furthermore, several studies demonstrated that propolis and its bioactive compounds may protect dermal collagen against environmental stressors, including UV-induced degradation, primarily through suppression of MMP-1 expression in dermal fibroblasts [41].

According to Hozzein et al. [37], the stimulation of collagen biosynthesis observed in the course of experimental diabetic wound healing is likely mediated by propolis-induced modulation of the TGF-β1/Smad2.3 signaling pathway as well as by the reduction of the levels of the pro-inflammatory cytokines IL-1β, IL-6, TNF-α, and MMP9 in the damaged tissue.

The beneficial effects of propolis-incorporated nonwoven dressings on the remodeling of ECM described in this study appear to be, at least in part, mediated by the stimulation of transforming growth factor-beta (TGF-β). This key regulatory molecule is essential during the early stages of wound repair, hemostasis, and inflammation [38]. It is important to emphasize that the therapeutic propolis impact is the result of the action of its diverse bioactive constituents, such as quercetin, caffeic and ferulic acid, apigenin, pinocembrin, or caffeic acid phenethyl ester (CAPE). The presence of the mentioned constituents in our propolis samples was definitively confirmed by the ESI-QTOF-MS analysis, as part of this study.

Among the identified compounds, quercetin significantly contributes to the downregulation of the pro-inflammatory cytokines such as TNF-α/IL-1β and the upregulation of IL-10. This shift facilitates the M1 to M2 macrophage phenotype transition, enhances fibroblast proliferation, and reduces chronic inflammation. Furthermore, quercetin increases the expression of VEGF and TGF-β, which are critical for angiogenesis and accelerated wound repair [42].

Similarly, vanillin acts as a bioactive agent that promotes angiogenesis, collagen deposition, re-epithelialization, biosynthesis of IL-10, and the TGF-β/VEGF gene expression, as well as IL-1β/TNF-α downregulation [58].

Another pluripotent molecule identified in our formulation is caffeic acid, which modulates myeloperoxidase and phospholipase A2 activity, reduces lipid peroxidation, and potently enhances the production of collagen-like polymers, as demonstrated in murine wound models [59].

Ferulic acid, another key bioactive molecule identified in the apitherapeutic nonwoven, contributes to tissue repair by reducing lipid peroxidation, stimulating catalase and superoxide dismutase activities, increasing glutathione concentration, supporting epithelialization, as well as the synthesis of hydroxyproline and hydroxylysine, which are essential for the stability of the collagen triple helix and subsequent cross-linking, vital for the biomechanical properties and integrity of the newly formed collagen network [60].

Another key active compound identified in the present study is apigenin. Previous research has demonstrated its potent antioxidant capacity, specifically in inhibiting lipid peroxidation. Apigenin promotes the accumulation of collagen fibers, enhances cellular viability, and mitigates tissue damage. Furthermore, it enhances angiogenesis, promotes fibroblast growth, modulates the expression of paracrine factors, notably through downregulation of TNF-α, IL-6, IL-1β, and the upregulation of VEGF [44,61].

The last two propolis constituents identified in this study by the ESI-QTOF-MS method are pinocembrin and caffeic acid phenethyl ester (CAPE).

Pinocembrin was shown to modulate macrophage activation and the synthesis of TNF-α, IL-1β, and IL-6; enhance IL-10 expression; and stimulate keratinocyte proliferation and survival by modulating the MAPK and PI3K pathways [62,63].

The second constituent, CAPE (Caffeic Acid Phenethyl Ester), has been shown to downregulate NF-κB expression, 5-lipoxygenase activity, and lipid hydroperoxide levels, while simultaneously upregulating glutathione levels, stimulating collagen accumulation, and supporting re-epithelialization in previous studies [64,65].

Importantly, CAPE, together with other propolis flavonoids, has also been reported to stabilize mast cells and inhibit their degranulation. Mast cells play a significant role in inflammatory and fibrotic responses during wound healing, and their excessive activation has been associated with abnormal scar formation. By reducing mast cell activation and the release of pro-inflammatory cytokines, propolis may contribute to improved collagen organization, reduced scar width, and more controlled tissue remodeling during the healing process [66,67,68,69,70].

Future studies should include gene expression analysis of key regulators of wound healing, such as collagen-related genes, angiogenic factors (e.g., VEGF), and signaling pathways involved in extracellular matrix remodeling, to provide deeper insight into the molecular mechanisms underlying the observed histological effects [38,40,42].

Taken together, the bioactive compounds identified in this study participate synergistically in tissue repair, contributing to a more organized histological pattern of tissue remodeling. Consequently, the propolis-incorporated nonwoven scaffolds utilized in our experiments appear promising as a biomaterial platform for supporting tissue organization in thermal skin injury repair. The disturbed repair process of tissue damage, often manifested by excessive fibrosis (scarring) or chronic inflammation, precludes the functional restoration of injured tissues and significantly prolongs patient convalescence. Consequently, the development of innovative therapeutic strategies—particularly for complex burn injuries—remains a critical priority. The propolis-incorporated scaffolds described in this study represent a promising approach in wound care, although their influence on overall clinical outcomes requires further confirmation in studies including macroscopic and quantitative endpoints.

### 3.1. Future Perspectives

Recent literature from 2024–2026 indicates that propolis-containing wound dressings are increasingly being developed as bioactive platforms capable of modulating the wound microenvironment rather than serving solely as passive protective barriers. This includes polysaccharide-based biomaterials functionalized with propolis, electrospun nanofibrous scaffolds, and core–shell delivery systems combining propolis with regenerative bioactive compounds [40,49,50,51,52,53,54].

In the context of the present findings, it may be hypothesized that PLGA–propolis scaffolds support favorable extracellular matrix remodeling in burn wounds. However, this hypothesis should be considered a direction for further investigation rather than definitive mechanistic evidence. In particular, future studies should combine AZAN-based histological assessment with molecular and immunohistochemical analyses, enabling differentiation of collagen subtypes and evaluation of cellular remodeling markers.

Future studies should therefore consider: immunohistochemical or immunofluorescent assessment of collagen type I and III; calculation of the collagen I/III ratio as an indicator of scar maturation and remodeling; evaluation of fibroblast and myofibroblast markers, including α-SMA, vimentin, and fibronectin; analysis of MMP-2, MMP-9, TIMP-1, and TIMP-2 to assess extracellular matrix turnover; investigation of the TGF-β/SMAD signaling pathway, which is strongly associated with collagen synthesis, fibroblast activity, and fibrosis; extension of angiogenesis assessment using CD31 or vWF markers; prospective histomorphometric analysis based on multiple randomly or systematically selected microscopic fields from each wound and each animal.

### 3.2. Limitations of the Study

Despite these promising findings, several limitations related to propolis application should be acknowledged. The chemical composition of propolis varies depending on geographical origin, botanical sources, and extraction methods, which may affect standardization, reproducibility, and consistency of therapeutic outcomes [71,72]. Additionally, propolis has been reported to induce allergic reactions, including contact dermatitis and hypersensitivity responses in susceptible individuals, which should be carefully considered in translational and clinical applications [73]. Furthermore, the immunogenicity of the propolis-incorporated scaffolds was not evaluated in the present study. Although no visible adverse local reactions were observed during the experimental period, further studies focusing on immunological responses and potential hypersensitivity reactions are required prior to clinical application.

A limitation of the present study is the lack of quantitative morphometric and statistical analysis of selected histological parameters. The study was designed primarily for qualitative, structural, and ultrastructural assessment; therefore, the findings should be interpreted within this descriptive framework. Future studies should incorporate prospectively defined quantitative image analysis and statistical evaluation to provide a more comprehensive assessment of the regenerative effects of propolis-based scaffolds. Another limitation of the present study is the lack of macroscopic documentation of wound healing, including gross wound images and quantitative analysis of wound closure over time. Therefore, no conclusions regarding wound closure rate or wound size reduction are drawn in this study. The study was primarily focused on microscopic evaluation of tissue structure and collagen organization, and therefore did not include assessment of wound size reduction dynamics. Future studies should incorporate macroscopic evaluation to better assess the overall therapeutic efficacy of propolis-based dressings.

Furthermore, the ultrastructural analysis did not include intact skin samples as a direct control for SEM and AFM imaging. Including such controls would have allowed for a more accurate comparison of collagen fibril organization and a better assessment of heat-induced structural changes. Therefore, future studies should incorporate imaging of intact tissues as a reference point for assessing changes in collagen architecture.

An additional limitation of the present study is the lack of formal randomization and blinding procedures. Although treatments were distributed across wounds in a standardized manner to minimize positional bias, the absence of random allocation and blinded outcome assessment may increase the risk of observer bias, particularly in qualitative histological evaluation. Future studies should incorporate randomized experimental design and blinded analysis to further enhance the methodological rigor and reliability of the results.

Moreover, the study did not include molecular or biochemical analyses (e.g., gene or protein expression), which would be required to directly confirm collagen type I/III expression.

Another limitation of the present study is the lack of mechanical characterization of the electrospun nonwovens. Although literature data suggest that PLGA-based scaffolds exhibit suitable mechanical properties for wound dressing applications, the influence of propolis incorporation on parameters such as tensile strength, elasticity, and structural stability was not directly assessed. This aspect should be addressed in future studies.

## 4. Materials and Methods

### 4.1. Materials

#### 4.1.1. Biological Material

The study protocol was approved by the Ethics Committee of the Medical University of Silesia in Katowice, Poland (no. LKE111/2014). All animal experiments were conducted at the Center for Experimental Medicine of the Medical University of Silesia. Four 16-week-old female domestic pigs (weighing 35–40 kg) were used in the study. The animals were maintained under standardized zoohygienic conditions to ensure optimal welfare and minimize stress throughout the experiment. All pigs received a complete R233 diet that fully met their nutritional requirements for energy, protein, amino acids, vitamins, and minerals. No additional supplementation that could influence the wound healing process was administered. Burn wounds were induced according to a standardized protocol based on the Hoekstra model [74], with additional procedural details provided below to ensure reproducibility. Prior to burn induction, the animals were premedicated with atropine sulfate (0.05 mg/kg body weight, s.c.), ketamine hydrochloride (3 mg/kg body weight, i.v.), and xylazine hydrochloride (1 mg/kg body weight, i.v.), followed by general anesthesia using thiopental sodium (5 mg/kg body weight, i.v.). After achieving deep anesthesia and analgesia, standardized full-thickness burn wounds were generated on the lateral surfaces of the animals’ bodies. Each animal received a total of 18 wounds (9 per side), symmetrically distributed, to ensure comparable experimental conditions. Burn injuries were created using a Lancetron D electrode heated to 170 °C, which was applied to the skin for 20 s under controlled pressure. Each wound measured 1.5 × 3.0 cm, ensuring uniform size and depth across all experimental groups.

Each animal was assigned to a single treatment condition (physiological saline, PLGA scaffold without propolis, PLGA scaffold with 5% propolis, or PLGA scaffold with 10% propolis). Within each animal, a total of 18 standardized burn wounds were generated (9 per side), corresponding to six predefined time points with three replicate wounds per time point. Thus, each experimental condition comprised 18 wound sites (*n* = 18), which were used for repeated qualitative assessment across time.

The allocation of treatments to wound sites was standardized and evenly distributed across anatomical locations to minimize potential positional bias. Each wound was treated as an independent experimental unit for histological evaluation.

Tissue samples were collected from the burn wound beds at predefined time points (days 3, 5, 10, 15, and 21 post-injury) depending on the experimental group (nonwoven PLGA dressings containing 5 wt% or 10 wt% propolis, sterile propolis-free nonwoven PLGA dressing, or physiological saline treatment). Control samples (day 0) were collected from the skin immediately after burn induction. All samples were immediately frozen and stored at −75 °C until further analysis.

Due to the qualitative nature of the study, no formal statistical analysis was performed. All tissue samples were collected using the same experimental model, standardized burn induction protocol, and predefined sampling time points across all experimental groups. Tissue processing, embedding, staining (AZAN method), and microscopic evaluation were performed under identical conditions for all samples, enabling direct qualitative comparison of histological features between groups. Representative microscopic fields were selected based on consistent morphological characteristics observed across multiple sections within each experimental group. In addition, a semi-quantitative analysis of selected histological parameters was performed as a supplementary assessment.

Reproducibility was ensured by applying a standardized experimental model, uniform wound induction parameters, and consistent sampling procedures across all experimental groups. Tissue processing, paraffin embedding, AZAN staining, and microscopic evaluation were performed under identical conditions for all samples. Representative microscopic fields were selected based on consistent morphological characteristics observed across multiple sections within each experimental group.

The experimental unit in this study was defined as an individual wound, with multiple wounds per animal assigned to different treatment groups.

#### 4.1.2. Propolis and Its Phytochemical Characterization

Propolis, also called “bee glue” in Eastern Europe, is collected by the Caucasian bee, *Apis mellifera caucasica*. The main sources of propolis obtained in the temperate zone are *Populus alba*, *Populus tremula*, *Populus nigra*, *Betula pendula*, *Alnus glutinosa*, *Pinus sylvestris*, and *Salix* sp. L. [19,41,71,72]. Popular propolis, supplied by Apipol-Farma (Myślenice, Poland), containing 50% plant resins and balms, 30% wax, 10% aromatic and essential oils, 5% pollen, and 5% additional active substances, was used in the described study.

The samples were prepared by dissolving propolis in methanol (concentration of 10 mg/mL), then filtered through the syringe membrane filter (0.4 um). The purified samples were analyzed qualitatively by an HPLC/ESI-QTOF-MS system in negative ion mode with the use of a 6530B Accurate-mass-QTOF-MS (Agilent Technologies, Inc., Santa Clara, CA, USA) mass spectrometer with an ESI-Jet Stream ion source. The Agilent 1260 chromatograph was equipped with a DAD detector, autosampler, binary gradient pump, and column oven. Gradient of solvents: water with 0.1% formic acid (solvent A) and acetonitrile with 0.1% formic acid (solvent B) were used as the mobile phases. The following gradient procedure was adopted: 0–45 min, 0–60% of B; 45–46 min, 60–95% B; 46–55 min, 95% B, the post time was 10 min.

The Phenomenex Gemini C18 3 μm 100 × 2 mm column was used as a stationary phase. Total time of analysis was 65 min, with a stable flow rate at 0.200 mL/min. The injection volume for the extracts was 10 μL. ESI-QToF-MS analysis was performed according to the following parameters of the ion source: Dual spray jet stream ESI, positive and negative ion mode, gas (N2) flow rate: 12 L/min., nebulizer pressure: 35 psig, vaporizer temp.: 300 °C; m/z range 100–1000 mass units, with acquisition Mode Auto MS/MS, collision induced dissociation (CID): 10 and 30 eV with MS scan rate 1 spectrum per s, 2 spectra per cycle, skimmer: 65 V, fragmentor: 140 V, and octopole RF Peak: 750 V.

Semiquantitative analyses were performed using the HPLC-DAD method. The Shimadzu high-performance liquid chromatograph (HPLC) was equipped with a diode array detector (DAD) SPDSM20A, a degasser DGU20A, a dual-channel gradient pump LC20AD, a column thermostat CTO10AS, and an autosampler SIL-20A. Zorbax Eclipse XDB C-18 (4.6 × 150 mm; dp = 3.5 µm) was used as the stationary phase. Water with 0.1% formic acid was used as mobile phase A, acetonitrile with 0.1% formic acid was used as mobile phase B. The following gradient was adopted: 0–40 min, 0–60% of B; 40–46 min, 60–95% B; 45–50 min, 95% (B), the post time was 10 min.

The flow rate of the mobile phase throughout the analysis was constant and maintained at 1 mL/min; the column was thermostated at 25 °C, sample injection was 10 µL, and the total time per analysis was 60 min. Data processing was performed in LabSolutions software (SHIMADZU CORPORATION (Tokyo, Japan)).

Quantitative analysis was based on two external standards, such as caffeic acid and quercetin (delivered by Sigma-Aldrich; Merck Life Science, Poznań, Poland). Analysis of main hydroxycinnamic derivatives, previously identified in the HPLC/MS method (caffeic acid, coumaric acid, ferulic acid, ethyl caffeate, dimethylallyl caffeate, and caffeic acid phenethyl ester (CAPE) was performed at 320 nm wavelength and counted as caffeic acid equivalent. Analysis of main flavonoids previously identified in the HPLC/MS method (quercetin, luteolin, isorhamnetin, pinobanksin, apigenin, pinostrobin, chrysin, and pinocembrin) was performed at 254 nm wavelength and counted as quercetin equivalent.

### 4.2. Methods

#### 4.2.1. Light Microscope (LM)

The samples of skin for histological analysis were fixed in Bouin’s solution at room temperature. At the next stage, tissues were rinsed in 80% ethyl alcohol and then embedded in paraffin blocks according to the standard histological procedure. Paraffin transversal sections of skin (6-µm thick) were stained by the AZAN trichrome method. The dried preparations were embedded in a synthetic resin DPX Mounting Medium (POCh, Gliwice, Poland), and analyzed using an Olympus BX60 microscope equipped with XC50 digital camera and Olympus cellSens Standard software. All histological sections were evaluated under the same imaging conditions to ensure consistency of qualitative morphological comparison between experimental groups.

As a supplementary assessment, semi-quantitative analysis was performed by manually counting the number of blood vessels and hair follicles in representative microscopic fields (*n* = 3 per group). Results are presented as mean ± standard deviation.

#### 4.2.2. Atomic Force Microscope (AFM)

AFM Measurement was performed on a NanoWizard^®^3 BioScience atomic force microscope (AFM) by JPK Instruments AG (Berlin, Germany), combined with a Zeiss Axio Observer (Jena, Germany) inverted optical microscope, which was utilized for all AFM measurements. The AFM was located in the laboratory with acoustic and vibration isolation to ensure absolute stability. The optical microscope with DIC contrast and maximum 400× magnification was used to aid the positioning of the AFM tip to the desired location on the surface of the skin cryosections. All experiments were performed in air using the intermittent contact mode (AC mode) with the Z range set to 15-µm and a scan rate of 0.5–1 Hz. Imaging was carried out using the Tapping Mode NSC15/AL BS etched silicon probes with a resonance frequency of 265–410 kHz (MikroMasch, Portland, OR, USA). The drive amplitude and set point were adjusted during measurement to minimize tip artifacts. The JPK SPM Software was used for AFM operation and later imaging processing. AFM usage allowed the elimination of the influence of the fixer and the obtaining of high-resolution images.

#### 4.2.3. Scanning Electron Microscope (SEM)

Transversal sections of skin were fixed in 2.5% glutaraldehyde and 1% osmium tetroxide solution in phosphate buffer at a pH = 7.4. After being washed several times using phosphate buffer solution, specimens were dehydrated with ethanol as follows: 30, 50, 70, 80, 90, and 96% for 15 min each. Following dehydration in the alcohol series and the CO2 critical point, the skin sections were gold-coated in a high-vacuum coater. The material was examined in a scanning electron microscope, Hitachi S-4700, at 20 kV.

#### 4.2.4. Nonwoven

Poly(lactide-co-glycolide) containing 85 mole-% of lactidyl and 15 mole-% of glycolidyl comonomeric units (PLGA 85:15) was synthesized according to the method described in the literature [75] in bulk via the ring opening polymerization (ROP) of l-lactide and glycolide. The reaction vessel was conditioned on an oil bath at 130 C for 24 h and then at 115 C for 72 h. The obtained copolymer was purified by dissolution in chloroform (Avantor Performance Materials Poland S.A., Gliwice, Poland) and precipitation into cold methanol (Avantor Performance Materials Poland S.A., Gliwice, Poland) to remove the unreacted monomers, followed by drying under a vacuum at room temperature. Solutions without active compound were prepared by dissolving the PLGA 85:15 copolymer (6% *w*/*w*) in 1,1,1,3,3,3-hexafluoro-2-propanol (HFIP) (Sigma Aldrich, Merck KGaA, Darmstadt, Germany). Solutions containing propolis were prepared by dissolving PLGA 85/15 (6% *w*/*w*) and dry propolis extract (Apipol-Farma, Myślenice, Poland) (5% (*w*/*w*) and 10% (*w*/*w*) propolis concentration relative to the amount of polymer) in HFIP (6% *w*/*w*). Solutions were used for obtaining nonwovens using the TL-Pro-BM electrospinning unit (Tong Li Tech, Shenzhen, China). Polymer solutions were dosed to the spinning nozzle through a capillary at 1.5 mL/h by using two Harvard Apparatus PHD Ultra 4400 (Harvard Apparatus, Cambridge, MA, USA) syringe pumps. The dosing volume was 22 mL. The temperature inside the chamber during electrospinning was 17.4 ± 0.8 °C, while the relative humidity was 43 ± 2%. Nonwovens were obtained in the form of 27 × 8.5 cm sheets. The procedure was repeated analogously for all solutions. The obtained nonwovens were dried under a vacuum at room temperature.

#### 4.2.5. Histological Image Analysis

Collagen-positive area fraction was determined using a custom Python (version 3.13.5, with OpenCV, NumPy, and Pillow libraries for color-threshold segmentation and mor-phological filtering) script applying HSV color-space thresholding supplemented by RGB channel-ratio conditions tuned to the blue-violet signal of AZAN staining. The binary mask was morphologically refined by median filtering, morphological opening, and removal of small connected components, and the denominator was restricted to tissue pixels with exclusion of background and label artifacts. The robustness of the observed trend was confirmed by sensitivity analysis comprising three independent thresholding variants (sensitive, standard, and strict).

#### 4.2.6. Scanning Electron Microscopy (SEM) of Electrospun Nonwovens

The morphology of nonwovens was analyzed by Surface Electron Microscope (Quanta 250 FEG, FEI Company, Hillsboro, OR, USA) operating in low vacuum conditions (80 Pa), with an acceleration voltage of 5 kV, from secondary electrons collected by Large Field Detector(FEI Company). Average diameters of fibers were calculated using ImageJ software (1.54r).

## 5. Conclusions

In conclusion, the experimental analysis of burns inflicted on pigs’ skin revealed distinct stages of tissue damage and subsequent structural tissue remodeling. The electrospun PLGA-based nonwoven fabric used provided a stable and biocompatible dressing, ensuring close contact with the wound surface throughout the healing period.

In the initial stage [wounds no. I. (tissue samples taken directly after burn and on postburn days 3rd and 21st)], burns caused superficial damage to the epidermis and dermis, with indications of collagen fibril alterations. In wounds no. I., on the third day post-burn, severe damage was evident, characterized by the destruction of epidermal and dermal cells, along with deficient collagen fibrils and visible empty spaces in the deeper dermal layer. By the twenty-first day, wounds no. I. exhibited significant damage in the superficial dermal layer, with limited collagen fiber staining, suggesting incomplete structural tissue remodeling. Histological signs of tissue organization [wounds no. II. (tissue samples taken on the 21st day after burn, treated with a sterile dressing)] included a keratinized stratified squamous epithelium in the epidermis and numerous bundles of collagen fibrils in the dermis. Intense staining with azocarmine indicated the presence of reticular fibers and budding blood vessels, reflecting the ongoing vascularization process. In wounds no. III. (21st day after burn, treated with 5% propolis nonwoven), pronounced histological features of tissue organization were observed, with a well-organized epidermis and dermis composed of abundant bundles of mainly collagen fibrils. Clear evidence of vascularization, along with regular structures of hair follicles, sebaceous glands, and blood vessels, highlighted the advanced stage of tissue recovery.

Furthermore, in wounds no. IV. (21st day after burn, treated with 10% propolis nonwoven), tissue organization was more advanced at the histological level compared with wounds no. III., with a regular skin structure and appropriate staining patterns using the AZAN method.

The presented SEM and AFM observations suggest that, in addition to its biological activity, the electrospun propolis-containing scaffold provides a mechanically favorable microenvironment that supports ordered collagen remodeling in burned tissue [10,14,16,29,31].

This comprehensive examination underscores the dynamic nature of the tissue response to burns, emphasizing the progressive stages of damage and structural tissue remodeling over the course of the experimental period.

## 6. Patents

In the experimental protocol, the apitherapeutic, biodegradable dressing protected by the patent (“Nonwoven dressing material and method of manufacturing nonwoven dressing material”, Date and patent number: 25 October 2021; 239827) has been applied.

## Figures and Tables

**Table 1:** Bioactive factors used.

Figures	Bioactive Factors	Skin Layer
Figure 2A	PLGA without propolis	epidermis
Figure 2A′	PLGA without propolis	dermis
Figure 2B	PLGA + 5% propolis	epidermis
Figure 2B′	PLGA + 5% propolis	ddermis
Figure 2C	PLGA + 10% propolis	epidermis
Figure 2C′	PLGA + 10% propolis	dermis

**Table 2:** Measurement effects of semi-quantitative analysis of representative observation areas of histological slides.

	Thresholding Sensitivity Analysis		
Group	Sensitive Mask (%)	Standard Mask (%)	Strict Mask (%)
II, PLGA without propolis	17.01	9.59	0.67
III, PLGA + 5% propolis	82.03	76.65	46.71
IV, PLGA + 10% propolis	67.88	62.94	39.82

**Table 4:** Semiquantitative results of the main hydroxycinnamic acid and flavonoid derivatives counted as caffeic acid and quercetin equivalents.

No.	Name	Concentration [mg/g]
1	Hydroxycinnamic acid derivatives (Caffeic acid equivalent)	5.52
2	Flavonoid derivatives (quercetin Equivalent)	45.70

## Data Availability

The data supporting the findings of this study are available within the article. Additional data are available from the corresponding authors upon reasonable request.

## References

[1] Wan X., Chen Y., Geng F., Sheng Y., Wang F., Guo J. (2022). Narrative Review of the Mechanism of Natural Products and Scar Formation in Wound Repair. Ann. Transl. Med..

[2] desJardins-Park H.E., Foster D.S., Longaker M.T. (2018). Fibroblasts and Wound Healing: An Update. Regen. Med..

[3] Zomer H.D., Trentin A.G. (2018). Skin Wound Healing in Humans and Mice: Challenges in Translational Research. J. Dermatol. Sci..

[4] Lee N., Bae Y., Jang S., Lee D.W., Lee S.W. (2025). Global, regional, and national burden of burn injury by total body surface area (TBSA) involvement from 1990 to 2021, with projections of prevalence to 2050. Healthcare.

[5] Siu W.S., Ma H., Leung P.C. (2025). Review on current advancements in facilitation of burn wound healing. Bioengineering.

[6] Stojko M., Wolny D., Włodarczyk J. (2021). Nonwoven Releasing Propolis as a Potential New Wound Healing Method—A Review. Molecules.

[7] Deng X., Gould M., Ali M.A. (2022). A review of current advancements for wound healing: Biomaterial applications andmedical devices. J. Biomed. Mater. Res. B Appl. Biomater..

[8] Zhao J., Chen L., Ma A., Bai X., Zeng Y., Liu D., Liu B., Zhang W., Tang S. (2024). Recent Advances in Coaxial Electrospun Nanofibers for Wound Healing. Mater. Today Bio.

[9] Zhang X., Wang Y., Gao Z., Mao X., Cheng J., Huang L., Tang J. (2024). Advances in wound dressing based on electrospinning nanofibers. J. Appl. Polym. Sci..

[10] Stojko M., Włodarczyk J., Sobota M., Karpeta-Jarząbek P., Pastusiak M., Janeczek H., Dobrzyński P., Starczynowska G., Orchel A., Stojko J. (2020). Biodegradable Electrospun Nonwovens Releasing Propolis as a Promising Dressing Material for Burn Wound Treatment. Pharmaceutics.

[11] Lu X., Zhou L., Song W. (2024). Recent progress of electrospun nanofiber dressing in the promotion of wound healing. Polymers.

[12] Tören E. (2026). Advancements in Portable Electrospinning Technology for Wound Healing Applications: A Comprehensive Review. Biomed. Mater. Devices.

[13] Winkler A., Maliszewska I., Czapka T. (2020). Elektroprzędzone Nanowłókna Polimerowe Do Zastosowań Medycznych. Polimery.

[14] Al-Enizi A.M., Zagho M.M., Elzatahry A.A. (2018). Polymer-Based Electrospun Nanofibers for Biomedical Applications. Nanomaterials.

[15] Mirjalili M., Zohoori S. (2016). Review for Application of Electrospinning and Electrospun Nanofibers Technology in Textile Industry. J. Nanostruct. Chem..

[16] Chattopadhyay S., Raines R.T. (2014). Review Collagen-Based Biomaterials for Wound Healing. Biopolymers.

[17] Olczyk P., Mencner Ł., Komosinska-Vassev K. (2014). The Role of the Extracellular Matrix Components in Cutaneous Wound Healing. BioMed Res. Int..

[18] Artem Ataide J., Caramori Cefali L., Machado Croisfelt F., Arruda Martins Shimojo A., Oliveira-Nascimento L., Gava Mazzola P. (2018). Natural Actives for Wound Healing: A Review. Phytother. Res..

[19] Wagh V.D. (2013). Propolis: A Wonder Bees Product and Its Pharmacological Potentials. Adv. Pharmacol. Sci..

[20] Woźniak M., Kwiatkowska A., Hołderna-Kędzia E., Sosnowska K., Mrówczyńska L., Ratajczak I. (2021). Aktywność biologiczna ekstraktów z propolisu. Postępy Fitoter..

[21] Woźniak M., Mrówczyńska L., Waśkiewicz A., Babicka M., Hołderna-Kędzia E., Ratajczak I. (2019). Zawartość Związków Fenolowych w Ekstrakcie z Propolisu Oraz Ocena Jego Aktywności Przeciwutleniającej i Cytoochronnej Względem Erytrocytów Ludzkich w Warunkach Stresu Oksydacyjnego in Vitro. Postępy Fitoter..

[22] Cabaj A., Juszczak L. (2021). Aktywność Przeciwutleniająca Handlowych Preparatów Propolisowych. Postępy Fitoter..

[23] Olczyk P., Mencner Ł., Komosinska-Vassev K. (2015). Diverse Roles of Heparan Sulfate and Heparin in Wound Repair. BioMed Res. Int..

[24] Olczyk P., Komosinska-Vassev K., Wisowski G., Mencner L., Stojko J., Kozma E.M. (2014). Propolis Modulates Fibronectin Expression in the Matrix of Thermal Injury. BioMed Res. Int..

[25] Olczyk P., Komosińska-Vassev K., Winsz-Szczotka K., Koźma E.M., Wisowski G., Stojko J., Klimek K., Olczyk K. (2012). Propolis Modulates Vitronectin, Laminin, and Heparan Sulfate/Heparin Expression during Experimental Burn Healing. J. Zhejiang Univ. Sci. B.

[26] Olczyk P., Komosinska-Vassev K., Winsz-Szczotka K., Stojko J., Klimek K., Kozma E.M. (2013). Propolis Induces Chondroitin/Dermatan Sulphate and Hyaluronic Acid Accumulation in the Skin of Burned Wound. Evid. Based Complement. Altern. Med..

[27] Olczyk P., Wisowski G., Komosinska-Vassev K., Stojko J., Klimek K., Olczyk M., Kozma E.M. (2013). Propolis Modifies Collagen Types I and III Accumulation in the Matrix of Burnt Tissue. Evid. Based Complement. Altern. Med..

[28] Olczyk P., Komosińska-Vassev K., Winsz-Szczotka K., Stojko J., Klimek K., Olczyk K. (2011). Wpływ Propolu T Na Akumulację Lamininy i Witronektyny w Macierzy Doświadczalnych Ran Oparzeniowych—Badania Wstępne. Farm. Pol..

[29] Rojczyk E., Klimek M., Wilemska-Kucharzewska K., Kucharzewski M. (2016). Rola Kolagenu w Procesie Gojenia Ran. Leczenie Ran.

[30] Aszódi A., Legate K.R., Nakchbandi I., Fässler R. (2006). What Mouse Mutants Teach Us about Extracellular Matrix Function. Annu. Rev. Cell Dev. Biol..

[31] Jiang Y., Lu S. (2014). Three-Dimensional Insights into Dermal Tissue as a Cue for Cellular Behavior. Burns.

[32] Daskalopoulos E.P., Janssen B.J.A., Blankesteijn W.M. (2012). Myofibroblasts in the Infarct Area: Concepts and Challenges. Microsc. Microanal..

[33] Summerfield A., Meurens F., Ricklin M.E. (2015). The immunology of the porcine skin and its value as a model for human skin. Mol. Immunol..

[34] Uhm C., Jeong H., Lee S.H., Hwang J.S., Lim K.M., Nam K.T. (2023). Comparison of structural characteristics and molecular markers of rabbit skin, pig skin, and reconstructed human epidermis for an ex vivo human skin model. Toxicol. Res..

[35] Rodrigues M., Kosaric N., Bonham C.A., Gurtner G.C. (2019). Wound Healing: A Cellular Perspective. Physiol. Rev..

[36] Landén N.X., Li D., Ståhle M. (2016). Transition from inflammation to proliferation: A critical step during wound healing. Cell. Mol. Life Sci..

[37] Hozzein W.N., Badr G., Al Ghamdi A.A., Sayed A., Al-Waili N.S., Garraud O. (2015). Topical application of propolis enhances cutaneous wound healing by promoting TGF-beta/Smad-mediated collagen production in a streptozotocin-induced type I diabetic mouse model. Cell. Physiol. Biochem..

[38] El-Sakhawy M., Salama A., Tohamy H.S. (2023). Applications of propolis-based materials in wound healing. Arch. Dermatol. Res..

[39] González-Masís J., Cubero-Sesin J.M., Corrales-Ureña Y.R., González-Camacho S., Mora-Ugalde N., Baizán-Rojas M., Loaiza R., Vega-Baudrit J.R., Gonzalez-Paz R.J. (2020). Increased Fibroblast Metabolic Activity of Collagen Scaffolds via the Addition of Propolis Nanoparticles. Materials.

[40] Koga H., Nishimura T., Kobayashi K. (2026). Effects of Brazilian green propolis and artepillin C on collagen metabolism and fibroblast behaviors: Implications for skin wound healing. Phytomedicine.

[41] Kim D.H., Auh J.H., Oh J., Hong S., Choi S., Shin E.J., Woo S.O., Lim T.G., Byun S. (2020). Propolis suppresses UV-induced photoaging in human skin through directly targeting phosphoinositide 3-kinase. Nutrients.

[42] Huang H.C., Chen Y., Hu J., Guo X.T., Zhou S.R., Yang Q.Q., Du Y.Q., Jin Y., Liu G.B., Peng Y.H. (2023). Quercetin and its derivatives for wound healing in rats/mice: Evidence from animal studies and insight into molecular mechanisms. Int. Wound J..

[43] Rojczyk E., Klama-Baryła A., Łabuś W., Wilemska-Kucharzewska K., Kucharzewski M. (2020). Historical and modern research on propolis and its application in wound healing and other fields of medicine and contributions by Polish studies. J. Ethnopharmacol..

[44] Rajab A.M., Al-Wattar W.T., Taqa G.T. (2022). The roles of apigenin cream on wound healing in rabbits model. J. Appl. Vet. Sci..

[45] Jastrzębska-Stojko Z., Stojko R., Rzepecka-Stojko A., Kabała-Dzik A., Stojko J. (2013). Biological activity of propolis-honey balm in the treatment of experimentally-evoked burn wounds. Molecules.

[46] Wang Y., Zheng L., Zhang L., Tai Y., Lin X., Cai Z. (2024). Roles of MMP-2 and MMP-9 and their associated molecules in the pathogenesis of keloids: A comprehensive review. Front. Pharmacol..

[47] Yigit E., Deger O., Korkmaz K., Huner Yigit M., Uydu H.A., Mercantepe T., Demir S. (2024). Propolis reduces inflammation and dyslipidemia caused by high-cholesterol diet in mice by lowering ADAM10/17 activities. Nutrients.

[48] Peixoto S., Nascimento A.P.S., Vicente C., Barros A.N. (2025). Solvent-Driven Extraction of Bioactive Compounds from Propolis for Application in Food Industry Matrices. Appl. Sci..

[49] Loya-Hernández L.P., Arzate-Quintana C., Castillo-González A.R., Camarillo-Cisneros J., Romo-Sáenz C.I., Favila-Pérez M.A., Quiñonez-Flores C.M. (2025). Propolis-Functionalized Biomaterials for Wound Healing: A Systematic Review with Emphasis on Polysaccharide-Based Platforms. Polysaccharides.

[50] El-Ghoul Y., Altuwayjiri A.S., Alharbi G.A. (2024). Synthesis and Characterization of New Electrospun Medical Scaffold-Based Modified Cellulose Nanofiber and Bioactive Natural Propolis for Potential Wound Dressing Applications. RSC Adv..

[51] Poodineh Hajipour F., Feyzbakhsh A., Maleknia L., Ahanian I. (2025). Electrospun Scaffold with Bioactive Polyurethane Shell Infused with Propolis and Starch-Hyaluronic Acid Core: An Advanced Therapeutic Platform for Skin Tissue Engineering. Int. J. Biol. Macromol..

[52] Orlińska K.M., Stocerz K., Kuczera M.A., Stojko M., Włodarczyk J., Kasperczyk J., Skalicka-Woźniak K., Kulinowski Ł., Tasinov O., Ivanova D. (2024). The Influence of Propolis Nonwoven Scaffolds on Burn Wound’s Heparan Sulfates and Hyaluronan. Appl. Sci..

[53] Elsamman M., El-Borady O.M., Nasr M.M., Al-Amgad Z., Metwally A.A. (2024). Development of Propolis, Hyaluronic Acid, and Vitamin K Nano-Emulsion for the Treatment of Second-Degree Burns in Albino Rats. BMC Complement. Med. Ther..

[54] Randall L.J., Bajan S., Tran T.D., Harvey R.J., Russell F.D. (2025). Propolis Compound Inhibits Profibrotic TGF-β1/SMAD Signalling in Human Fibroblasts. Sci. Rep..

[55] Eskandarinia A., Kefayat A., Gharakhloo M., Agheb M., Khodabakhshi D., Khorshidi M., Sheikhmoradi V., Rafienia M., Salehi H. (2020). A propolis enriched polyurethane-hyaluronic acid nanofibrous wound dressing with remarkable antibacterial and wound healing activities. Int. J. Biol. Macromol..

[56] Yang J., He Y., Nan S., Li J., Pi A., Yan L., Xu J., Hao Y. (2023). Therapeutic effect of propolis nanoparticles on wound healing. J. Drug Deliv. Sci. Technol..

[57] Sharaf S.M., Al-Mofty S.E., El-Sayed E.M., Omar A., Abo Dena A.S., El-Sherbiny I.M. (2021). Deacetylated cellulose acetate nanofibrous dressing loaded with chitosan/propolis nanoparticles for the effective treatment of burn wounds. Int. J. Biol. Macromol..

[58] Arya S.S., Rookes J.E., Cahill D.M., Lenka S.K. (2021). Vanillin: A review on the therapeutic prospects of a popular flavouring molecule. Adv. Tradit. Med..

[59] Song H.S., Park T.W., Sohn U.D., Shin Y.K., Choi B.C., Kim C.J., Sim S.S. (2008). The effect of caffeic acid on wound healing in skin-incised mice. Korean J. Physiol. Pharmacol..

[60] Zduńska K., Dana A., Kołodziejczak A., Rotsztejn H. (2018). Antioxidant properties of ferulic acid and its possible application. Skin Pharmacol. Physiol..

[61] Ma X., Lin Y., Liu Y., Li W., He J., Fang M., Lin D. (2021). Effects of Apigenin Treatment on Random Skin Flap Survival in Rats. Front. Pharmacol..

[62] Elbatreek M.H., Mahdi I., Ouchari W., Mahmoud M.F., Sobeh M. (2023). Current advances on the therapeutic potential of pinocembrin: An updated review. Biomed. Pharmacother..

[63] Ruttanapattanakul J., Wikan N., Potikanond S., Nimlamool W. (2022). Molecular Targets of Pinocembrin Underlying Its Regenerative Activities in Human Keratinocytes. Pharmaceuticals.

[64] Serarslan G., Altuğ E., Kontas T., Atik E., Avci G. (2007). Caffeic acid phenethyl ester accelerates cutaneous wound healing in a rat model and decreases oxidative stress. Clin. Exp. Dermatol..

[65] Romana-Souza B., Dos Santos J.S., Monte-Alto-Costa A. (2018). Caffeic acid phenethyl ester promotes wound healing of mice pressure ulcers affecting NF-κB, NOS2 and NRF2 expression. Life Sci..

[66] Barroso P.R., Lopes-Rocha R., Pereira E.M.F., Marinho S.A., de Miranda J.L., Lima N.L., Verli F.D. (2012). Effect of propolis on mast cells in wound healing. Inflammopharmacology.

[67] Chirumbolo S. (2012). Flavonoids in propolis acting on mast cell-mediated wound healing. Inflammopharmacology.

[68] Wilgus T.A., Wulff B.C. (2014). The importance of mast cells in dermal scarring. Adv. Wound Care.

[69] Wilgus T.A., Ud-Din S., Bayat A. (2020). A Review of the Evidence for and against a Role for Mast Cells in Cutaneous Scarring and Fibrosis. Int. J. Mol. Sci..

[70] Chen L., Schrementi M.E., Ranzer M.J., Wilgus T.A., DiPietro L.A. (2014). Blockade of mast cell activation reduces cutaneous scar formation. PLoS ONE.

[71] Ballouk M.A.-H., Altinawi M., Fudalej P.S. (2025). The Multifaceted Therapeutic Potential of Propolis: An Integrative Report on Its Pharmacological Properties and Emerging Advances. Discov. Appl. Sci..

[72] Alsarayrah N.A., Mohamed R., Omar E.A. (2025). Stingless Bee Propolis: A Comprehensive Review of Chemical Constituents and Health Efficacy. Nat. Prod. Bioprospecting.

[73] Nyman G., Oldberg Wagner S., Prystupa-Chalkidis K., Ryberg K., Hagvall L. (2020). Contact Allergy in Western Sweden to Propolis of Four Different Origins. Acta Derm. Venereol..

[74] Hoekstra M.J., Hupkens P., Dutrieux R.P., Bosch M.M.C., Brans T.A., Kreis R.W. (1993). A Comparative Burn Wound Model in the New Yorkshire Pig for the Histopathological Evaluation of Local Therapeutic Regimens: Silver Sulfadiazine Cream as a Standard. Br. J. Plast. Surg..

[75] Dobrzynski P., Kasperczyk J., Janeczek H., Bero M. (2001). Synthesis of Biodegradable Copolymers with the Use of Low Toxic Zirconium Compounds. 1. Copolymerization of Glycolide with l-Lactide Initiated by Zr(Acac)4. Macromolecules.

